# Evidence for dimerization of ferroportin in a human hepatic cell line using proximity ligation assays

**DOI:** 10.1042/BSR20191499

**Published:** 2020-05-05

**Authors:** Gautam Rishi, Eriza S. Secondes, Daniel F. Wallace, V. Nathan Subramaniam

**Affiliations:** Institute of Health and Biomedical Innovation and School of Biomedical Sciences, Queensland University of Technology (QUT), Brisbane, Queensland, Australia

**Keywords:** ferroportin disease, Ferroportin, iron homeostasis, iron overload, proximity ligation assays

## Abstract

Mutations in the only known iron exporter ferroportin (FPN) in humans are associated with the autosomal dominantly inherited iron overload disorder ferroportin disease or type IV hereditary hemochromatosis (HH). While our knowledge of the central role of FPN in iron homeostasis has grown in the last 20 years, there exist some questions surrounding the structure and membrane topology of FPN with conflicting data on whether this receptor acts as a monomer or a multimer. To investigate and determine if FPN dimerization occurs in cells, we used novel tools including a variety of different FPN constructs expressing different tagged versions of the protein, a novel antibody that only detects cell surface FPN and proximity ligation assays. The results of the present study suggest that both the carboxy- and amino-termini of the FPN protein are intracellular. We also show that exogenously transfected FPN forms dimers; these dimers can be formed between the wild-type and mutant FPN proteins. This is the first study to examine the intracellular dimerization of FPN protein. Using proximity ligation assays, we show intracellular localization of FPN dimers and the interaction between FPN and hepcidin proteins as well. These results have important implications in the field of iron metabolism and add to our knowledge about FPN membrane topology and physiology of iron transport. This will be of importance in understanding the clinical implications of FPN mutations and of interest to future research aimed at targeting FPN expression to modulate iron homeostasis.

## Introduction

Systemic iron levels are maintained by regulating the amount of iron absorbed by the body through enterocytes and in recycling of iron from senescent red blood cells by macrophages. This is maintained by the proper interaction between the iron hormone, hepcidin, and the iron exporter, ferroportin (FPN). FPN is expressed on the surface of most cells and tissues involved in systemic iron homeostasis, including enterocytes, hepatocytes, placental tissue and macrophages. In conditions of increased body iron levels, the hepatocytes produce hepcidin, which then binds to surface-expressed FPN leading to its internalization and degradation [1,2]. A reduction in the amount of surface FPN protein means the cells are unable to export iron, leading to a reduction of iron being released into the blood from the sites of absorption (enterocytes), recycling (macrophages) or storage (hepatocytes).

FPN is the only known iron exporter in mammals and the hepcidin–ferroportin axis is central to maintaining iron homeostasis. The role of hepcidin and its regulation has been the focus of many reviews [3–5], which discuss the various stimuli that can regulate hepcidin, including body iron levels, inflammation and others. FPN itself can be regulated at multiple levels: transcriptional, post-transcriptional and post-translational [6]. The absence of a regulated mechanism to get rid of the excess iron adds importance to the role FPN plays in maintaining iron levels. Three different groups using completely different approaches discovered FPN almost simultaneously [7–9]. Since its discovery, we and others have identified a number of mutations in the gene encoding FPN (*SLC40A1*) associated with iron overload [10–25]. Mutations in FPN can be classified into two different types [6]. First, loss-of-function mutations that impair the iron export ability of the protein, often referred to as ‘Ferroportin Disease’ (FD) [6]. The second class of mutations result in the protein either not binding to hepcidin or where binding is inconsequential and FPN is able to constitutively export iron; these mutations do not affect the iron export ability of the protein. Hemochromatosis caused by the second class of mutations is pathologically similar to other forms of hereditary hemochromatosis, which is more aptly known as ‘Ferroportin-associated hemochromatosis’ [6].

Although we are certain about the function of the protein, there have been controversies regarding the membrane structure and the molecular organization of the protein. Studies have suggested varying numbers of transmembrane regions in the protein; based on computational modelling, we and others have suggested that the full-length FPN protein that is made up of 12 transmembrane regions [26,27]. Another controversy has been the localization of the amino (N)- and carboxy (C)-termini of the proteins, with studies suggesting different possibilities [24,26,28–31]. Based on the number of transmembrane regions that we and others proposed, it was suggested that both the N- and C-termini are intracellular and only the transmembrane regions are exposed on the surface of the cells [26].

The other major controversy is the question of whether FPN acts as a monomeric or a multimeric protein [6]. In studies using exogenous tagged FPN protein, it has been shown that FPN forms multimers and that the mutant FPN prevents cell surface localization of the wild-type (WT) protein [32,33]. Whereas, other studies using similar methods have suggested that FPN does not form multimers [27,34,35]. The majority of mutations reported so far for FD are missense variants [12,36], which support the dominant-negative effect that would be expected in the case of the protein forming multimers. Similarly, a gene deletion in the mouse *Fpn* gene has little to no effect on the iron status of heterozygous mice [37].

A recent study used crystal structures of the bacterial homolog of FPN to predict the structure of the human FPN protein [29]. According to the proposed model, FPN has 12 transmembrane helices and is divided into two halves forming the N and the C lobes. The cavity between these two lobes undergoes a conformational change to allow iron export, but the binding site is only accessible when the cavity opens up toward the extracellular region [29]. In this open configuration, hepcidin is able to bind to FPN and induce the ubiquitination and degradation of the protein. This model supports the view that the human FPN protein can function as a monomer, but is based on the structure of a prokaryotic protein [29]. A more recent model was proposed by Sabelli et al. who used patient-derived macrophages from ferroportin disease patients [38]. The authors showed that FPN does not form multimers in the patient macrophages and the protein is able to function normally in the cells, where it is localized to the cell membrane, can export iron and also responds to increases in hepcidin, which leads to the internalization and degradation of FPN protein [38]. In conditions of excess iron, the localization of the FPN protein to the surface is reduced and thus leads to increased iron retention in the cells [38]. This model explains the function and mutational effect of FPN acting as a monomer; however, the study did not examine the differences in the localization of mutant and WT FPN protein in the same cells and also if the mutants were able to transport iron as effectively as the WT molecules. These contradictory results and the inability to explain some of the observed phenotypes prompted us to revisit the issue of FPN dimerization utilizing the novel resources available to us.

In the present study, we examined whether (a) the C- and N-termini of the FPN protein are intracellular or extracellular, (b) the tagged version of FPN is iron export competent, (c) if FPN forms a dimer irrespective of being a WT or mutant protein and (d) whether we can detect cis-interactions between hepcidin and FPN when expressed in the same cell. In order to answer these questions, we utilized novel antibodies generated in our laboratory and the proximity ligation assay to study the potential molecular interactions between FPN homodimers and between FPN and hepcidin. Using various tagged versions of WT and mutant FPN, we were able to demonstrate that both the C- and N-termini of FPN protein are intracellular and that overexpressed FPN protein forms dimers in the hepatocyte cell line HuH7. We also show that FPN and hepcidin can bind to each other and these interactions can be detected within the same cell.

## Materials and methods

### Generation of FPN constructs

The WT FPN constructs with C-terminal myc tag (C-myc-FPN), N-terminal myc tag (N-myc-FPN) and p.V162del FPN (C-myc-FPN-V162) were described previously [26]. The plasmid constructs expressing WT and p.C326Y FPN with HA tag were described in [12] and the hepcidin construct was described in [39]. The plasmid construct expressing WT FPN with a Flag tag was kindly provided by Professor Hal Drakesmith (University of Oxford).

### Cell culture and transfections

The human hepatoma cell line HuH7 was obtained from ATCC (American Type Culture Collection, Manassas, VA) and cultured in RPMI with 10% fetal calf serum (FCS) in 25 cm^2^ flasks. Transfections were performed using Lipofectamine 3000 reagent according to the manufacturer’s instructions (Invitrogen, Mulgrave, Victoria, Australia). Cells were seeded at a density of 200,000 cells in a six-well plate. A total of 1 µg plasmid DNA was used for all transfections: 1 µg for single transfections and 500 ng of each plasmid for double transfections.

### Ferritin assay

The cells were seeded on collagen-coated glass coverslips for 24 h before treating with 100 µM ferric ammonium citrate (FAC). After 24 h treatment, the cells were transfected with FPN plasmid constructs as explained above. After 24 h, the cells were fixed and used for immunofluorescence analysis.

### Immunofluorescence

Cells were seeded on collagen-coated glass coverslips and grown to a confluence of 75–80%. After washing with PBSCM (PBS, 1 mM CaCl_2_, 1 mM MgCl_2_), the cells were fixed with 3% paraformaldehyde (PFA) for 10 min at room temperature (RT). The fixed cells were then washed with 50 mM NH_4_Cl to quench the PFA, followed by a PBSCM wash, then permeabilized with 0.1% saponin in PBSCM for 10 min at RT and incubated with primary antibodies. The primary antibodies used in the present study were: rabbit anti-HA (1:100) (Cell Signalling Technologies, Danvers, MA), mouse anti-FLAG M2 (1:1000) (Merck, Merck, Frenchs Forest NSW, Australia), rabbit anti-ferritin (1:1000) (CST), rabbit anti-myc (1:100) (CST) and rabbit anti-Floopn (1.3 µg/ml) [12] diluted in fluorescence dilution buffer (FDB) (5% fetal calf serum, 5% normal donkey serum, 2% bovine serum albumin in PBSCM, pH 7.6) for 1 h at RT. After the primary antibody incubation, the excess antibody was washed off using PBSCM and the cells were incubated with the secondary antibodies as described below.

For surface staining, the cells were washed with PBSCM, then incubated with primary antibodies for 1 h. After primary antibody incubation, the cells were washed with PBSCM and fixed with 3% PFA for 10 min at RT. The PFA was quenched with 50 mM NH_4_Cl followed by a PBSCM wash and the cells were then incubated with secondary antibodies. Donkey anti-mouse Alexa488 and donkey anti-rabbit Alexa594 (Invitrogen) were diluted in FDB and incubated for 1 h at RT. After washing with PBSCM, the coverslips were then mounted using Prolong Gold anti-fade with DAPI (Invitrogen). The imaging and visualization of the fluorescently stained cells was performed using the Zeiss 710 confocal microscope and the image analysis was performed using the Zeiss software.

### Proximity ligation assays

The cells were seeded on 15 mm collagen-coated coverslips for this experiment and transfected as described above. The mouse/rabbit starter Duolink kit (Merck) was used as described [40]. The antibody concentrations used for this assay were the same as was used for the immunofluorescence assay described above. In addition, we used mouse anti-Tfr1 (1:500) (Zymed, Thermo Fischer Scientific, Seventeen Mile Rocks, QLD, Australia) and rabbit anti-Tfr1 (1:100) (Abcam, Cambridge, U.K.) as positive controls for the experiment.

### Western blotting

HuH7 cells were seeded in six-well plates at a cell density of 300,000 cells per well for 24 h prior to treating with 100 µM FAC. After a further 24 h, transfections with various FPN expressing constructs were performed. The cells were lysed in 1× Laemmli sample buffer and electrophoresed on a 12% SDS-PAGE followed by Western blotting as previously described [41]. The blot was then incubated with anti-ferritin (1:5000) and anti-actin (1:10,000) (Merck) overnight at 4°C. After washing with 10% Tween 20 in Tris-buffered saline (TBST), the blots were incubated with anti-rabbit horseradish peroxidase (Thermo-Fisher) for 1 h. The blots were then developed using a Minolta film developer after incubating with substrate (Luminata Forte (Merck)) for 5 min using X-ray films (Fuji films). The scanned images were quantitated using ImageJ software.

## Results

### Exogenously expressed tagged versions of FPN protein are functional

**Figure 1:**
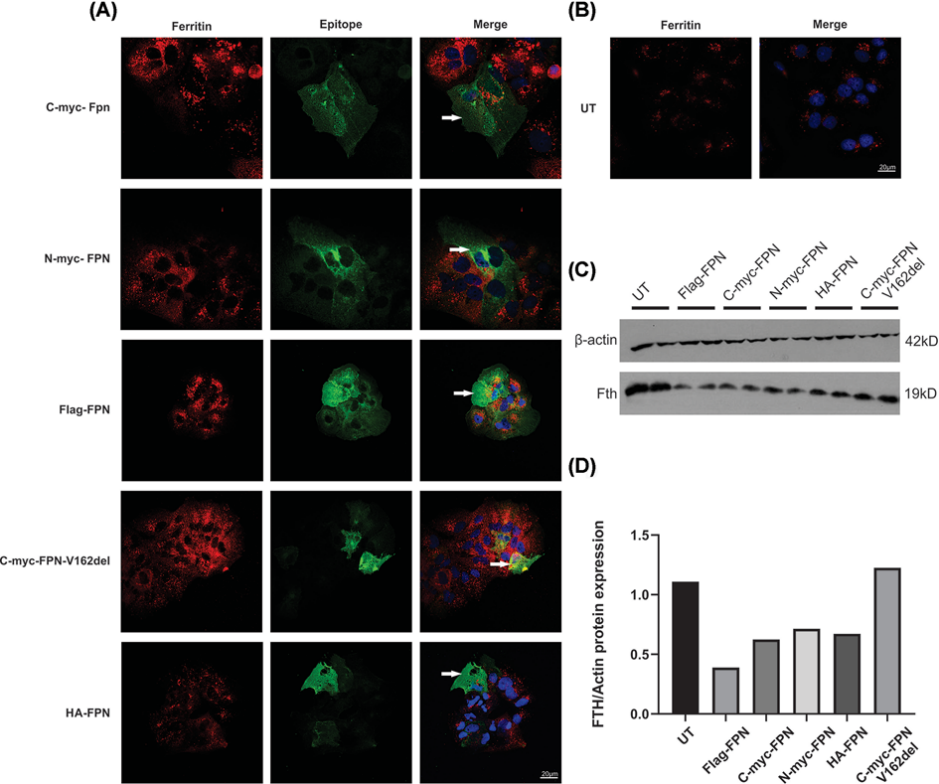
Exogenously expressed and epitope tagged versions of FPN are iron export competent (**A**) Immunofluorescence microscopy was performed on HuH7 cells treated with 100 µM FAC for 24 h and transfected with wild-type FPN constructs with C-terminal myc tag (C-myc-FPN), N-terminal myc tag (N-myc-FPN), Flag-tag (Flag-FPN), p.V162del FPN mutant (C-myc-FPN-V162del) and HA-tag (HA-FPN). The expression of FPN was detected using antibodies against the corresponding epitope tag (green, middle panel), and iron was detected using the ferritin antibody (red, left panel). All the WT FPN proteins were able to export iron efficiently (shown by absence of red staining for ferritin, in cells expressing FPN in green), whereas the p.V162del FPN mutant is unable to export iron efficiently as shown by the presence of red and green staining in the same cell; scale bar = 20 µM. (**B**) Immunofluorescence on untreated HuH7 cells showing low basal levels of endogenous iron as shown in red, detected using a ferritin antibody; scale bar = 20 µM. (**C**) Western blot showing a decrease in ferritin expression in HuH7 cells treated with 100 µM FAC for 24 h and transfected with wild-type FPN constructs with Flag-tag (Flag-FPN), C-terminal myc tag (C-myc-FPN), N-terminal myc tag (N-myc-FPN), HA-tag (HA-FPN), while there was no decrease in ferritin expression in the cells transfected with p.V162del FPN mutant (C-myc-FPN-V162del). (**D**) Quantitation of the Western blot shown in panel (C) shows a decrease in ferritin expression in the cells transfected with WT-FPN (Flag-FPN, C-myc-FPN, N-myc-FPN and HA-FPN), whereas the mutant (C-myc-FPN-V162del) had no decrease in ferritin.

HuH7 cells were transfected with different constructs of FPN (WT and mutant, p.V162del) tagged with either myc, FLAG or HA epitope tags. We have previously shown that the p.V162del FPN mutant can be transported to the surface but is iron export deficient [26]. We determined the effect of different tags on the ability of the FPN protein to export iron. The cells were treated with 100 µM FAC for 24 h to load them with iron and then transfected with either WT C-myc-FPN, N-myc-FPN, Flag-FPN, HA-FPN or the mutant C-myc-FPN-V162del. After 24 h, the cells were then stained to detect ferritin (as a marker of endogenous iron levels) and FPN (using antibodies against the various epitope tags). We have previously used this assay to determine whether the FPN protein is iron-export competent [26]. All the WT FPN proteins were able to export iron effectively (Figure 1A,C,D), irrespective of the epitope tags, as shown by the absence of ferritin staining (red) in cells expressing FPN (green). The minimal red staining in the untreated Huh7 cells (Figure 1B) suggest that the ferritin increase in the treated cells is due to FAC treatment. As shown previously [26], the ability of mutant FPN (C-myc-FPN-V162del) to export iron is compromised (Figure 1A), which shows ferritin and FPN in the same cell (red and green staining). The Western blotting with ferritin antibody also confirms that the WT FPN, irrespective of the tag, can efficiently transport iron (Figure 1C,D) as shown by the decrease in ferritin expression, whereas the p.V162del FPN mutant had no decrease in ferritin. These results demonstrate that all the WT FPN constructs used in the present study expressed functional FPN protein.

### Membrane topology of FPN protein

**Figure 2:**
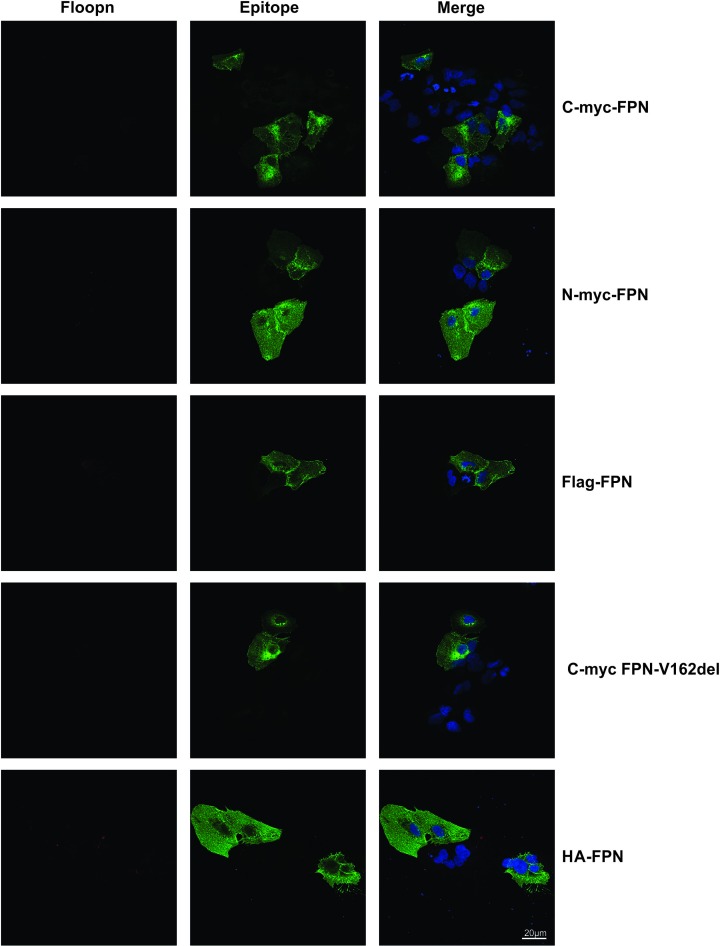
Intracellular FPN expression by confocal microscopy Immunofluorescence microscopy was performed on HuH7 cells transfected with wild-type FPN constructs with C-terminal myc tag (C-myc-FPN), N-terminal myc tag (N-myc-FPN), Flag-tag (Flag-FPN), p.V162del FPN mutant (C-myc-FPN-V162del) and HA-tag (HA-FPN). Intracellular immunofluorescence was performed using either the Floopn antibody (left panel) or the epitope tag specific antibody (green staining, middle panel). Both the C-and N-termini of FPN protein can be detected using this method as shown by the presence of green staining in the middle panel; scale bar = 20 µM.

**Figure 3:**
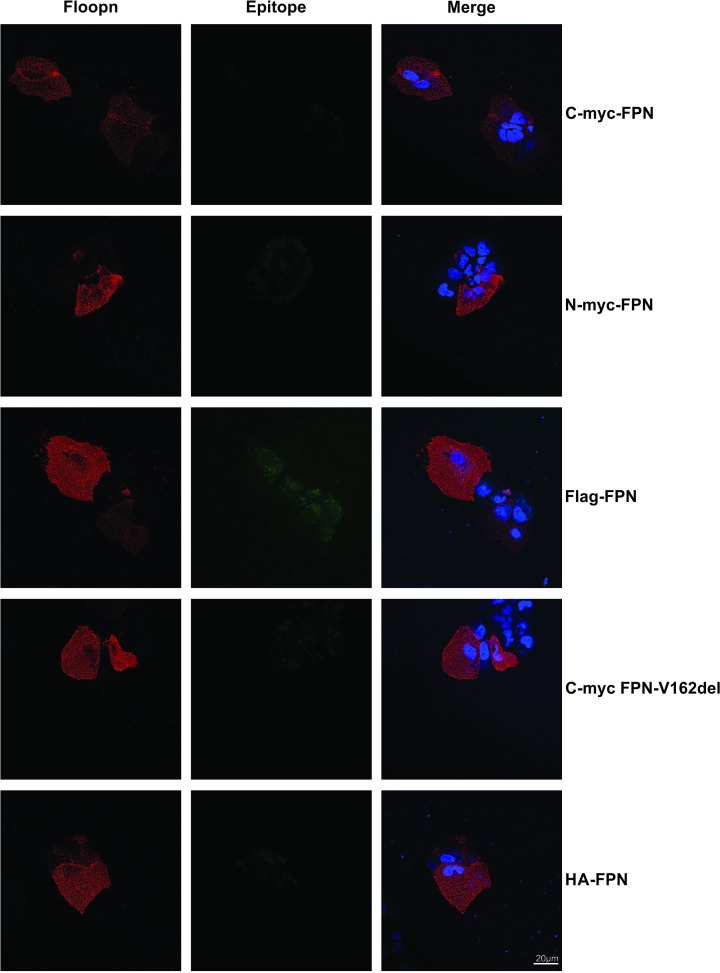
Surface expression of FPN by confocal microscopy Immunofluorescence microscopy was performed on HuH7 cells transfected with wild-type FPN constructs with C-terminal myc tag (C-myc-FPN), N-terminal myc tag (N-myc-FPN), Flag-tag (Flag-FPN), p.V162del FPN mutant (C-myc-FPN-V162del) and HA-tag (HA-FPN). Surface immunofluorescence was performed using either the Floopn antibody (red staining, left panel) or the epitope tag specific antibody (middle panel). The absence of any extracellular/surface immunofluorescence signal with the tag-specific antibodies suggests that both the C-and N-termini of FPN are intracellular; scale bar = 20 µM.

We then examined the localization of the N- and C-termini of the FPN protein. To do this, we used a novel antibody specific for the loop between transmembrane (TM) helices 9 and 10 of human FPN, to examine surface staining of FPN generated in our laboratory (termed Floopn) [12]. We performed co-localization studies using the Floopn antibody and epitope tag antibodies. The epitope tag-specific antibodies were able to detect intracellular FPN staining (green staining, Figure 2, middle panel), whereas we could not detect any cell surface staining with these antibodies (Figure 3, middle panel). Similarly, the Floopn antibody was able to detect surface expression (red staining, Figure 3, left panel) in all cells transfected with FPN constructs, irrespective of the tags, showing that the epitope-tagged FPN does traffick to the cell surface. These results confirm that both the N- and C-termini of the FPN protein are expressed intracellularly. Interestingly, the Floopn antibody was unable to detect any intracellular expression of the FPN protein (Figure 2, left panel).

### Proximity ligation assay detects homodimer forms of WT and mutant FPN and interactions with hepcidin

**Figure 4:**
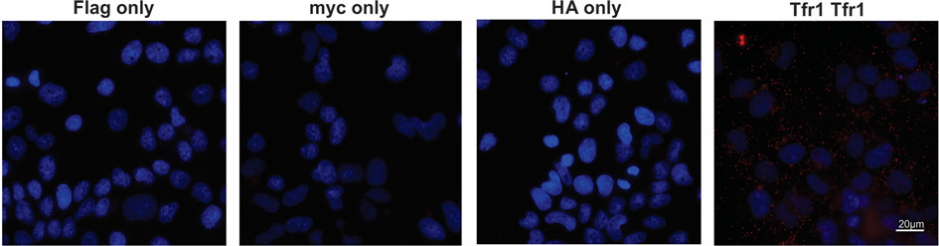
Single antibody incubation controls for proximity ligation assays demonstrate specificity of assay Proximity ligation assays were performed on HuH7 cells transfected with wild-type FPN constructs with Flag-tag (Flag-FPN), C-terminal myc tag (C-myc-FPN) and HA-tag (HA-FPN). The non-specific signal was examined by incubating transfected HuH7 cells with single antibodies directed against Flag, myc or HA epitope tags only. The absence of any red dots in any of the first three panels suggests that the assay is specific. The formation of TFR1 homodimers (panel 4) was used as a positive control. To detect the formation of dimers, antibody combinations against the corresponding epitopes were used in each experiment (TFR1 antibodies for TFR1 homodimers and Flag, myc and HA antibodies for FPN dimers). The presence of red dots in each of the panels suggests that FPN can form dimers and that TFR1 forms homodimers as shown previously [40]; scale bar = 20 µM.

**Figure 5:**
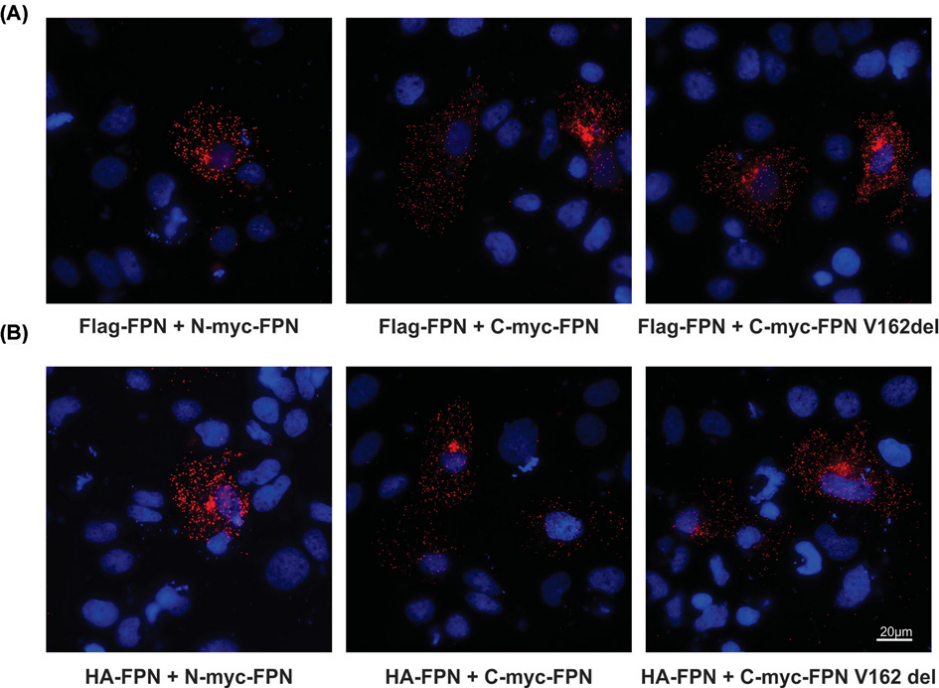
Proximity ligation assay indicates FPN-FPN dimer formation (**A**) PLA was performed on HuH7 cells co-transfected with Flag-tag (Flag-FPN) and wild-type FPN construct with N-terminal myc tag (N-myc-FPN), Flag-tag (Flag-FPN) and wild-type FPN construct with C-terminal myc tag (C-myc-FPN) or Flag-tag (Flag-FPN) and p.V162del FPN mutant (C-myc-FPN-V162del). (**B**) PLA was performed on HuH7 cells co-transfected with HA-tag (HA-FPN) and wild-type FPN construct with N-terminal myc tag (N-myc-FPN), HA-tag (HA-FPN) and wild-type FPN construct with C-terminal myc tag (C-myc-FPN) or HA-tag (HA-FPN) and p.V162del FPN mutant (C-myc-FPN-V162del). The formation of bright red spots in all these panels suggests that these differentially tagged forms of FPN, whether WT or mutant are able to form dimers; scale bar = 20 µM.

**Figure 6:**
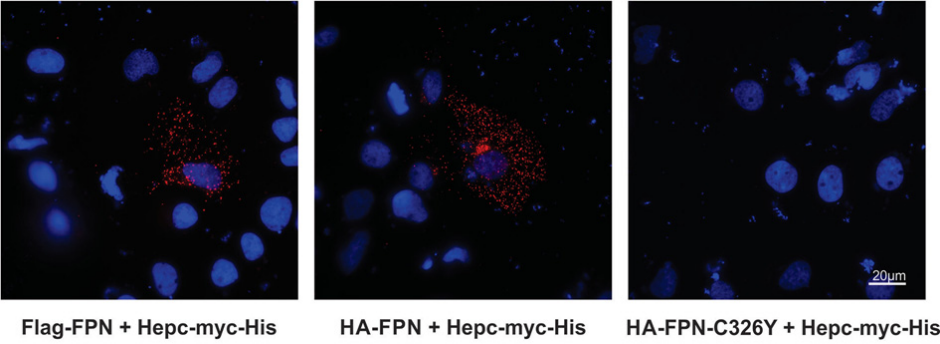
Proximity ligation assay to show FPN-hepcidin interactions PLA was performed on HuH7 cells co-transfected with either plasmids expressing WT-FPN (Flag-FPN, HA-FPN) or p.C326Y FPN mutant (HA-FPN-C326Y) with WT hepcidin (Hepc-myc-His). To detect the interactions, antibody combinations against the corresponding epitopes (Flag, HA or myc) were used. The presence of red dots in the first two panels suggests that PLA can be used to detect interactions between WT FPN and hepcidin, whereas the p.C326Y FPN mutant does not interact with hepcidin as shown by the absence of red spots in the third panel.

We then examined whether FPN forms homodimers. To this effect, HuH7 cells were transfected with combinations of different FPN constructs, either C-myc-FPN and Flag-FPN, N-myc-FPN and Flag-FPN or C-myc-FPN-V162del and Flag-FPN. We also transfected cells with different combinations with HA-FPN co-transfected wild-type FPN construct with N-terminal myc tag (N-myc-FPN), HA-tag (HA-FPN) and wild-type FPN construct with C-terminal myc tag (C-myc-FPN) or HA-tag (HA-FPN) and p.V162del FPN mutant (C-myc-FPN-V162del). Previous studies have used conventional co-immunoprecipitation (co-IP) experiments in order to determine whether the FPN molecules form multimers; in order to visualize these interactions *in situ*, we utilized proximity ligation assays (PLA). PLA is a technique that is increasingly used to detect, visualize, localize and quantitate the number of interactions between two proteins of interest at a molecular level in their native forms inside cells and tissues. PLA was performed on doubly transfected cells 24 h post-transfection. To demonstrate the specificity of the assay, PLA was performed with single antibodies and as shown in Figure 4, we did not observe any staining (red spots) when only one of the antibodies was used for PLA (panels 1–3), suggesting that any interactions observed are most likely to be specific. In this assay, we could detect TFR1-TFR1 dimers (Figure 4, panel 4) that served as a positive control for the assay, as we have previously shown [40]. Results from the PLA (Figure 5) showed that Flag-FPN and N-myc-FPN, Flag-FPN and C-myc-FPN, or Flag-FPN and C-myc-FPN-V162del were all able to form dimers in HuH7 cells transfected with the respective constructs (Figure 5A). Similarly, the presence of red spots in cells transfected with HA-FPN and N-myc-FPN, HA-FPN and C-myc-FPN, or HA-FPN and C-myc-FPN-V162del shows that these versions of FPN can also form dimers (Figure 5B). In addition, we also examined whether we could detect the interaction between FPN and its interacting peptide, hepcidin, in these cells. Cells were transfected with either Hepc-myc-His [39] and Flag-FPN, HA-FPN or HA-FPN-C326Y. The C326Y mutant has previously been shown to be hepcidin insensitive, and in the present study, we show that although WT FPN binds with hepcidin (shown by the red dots), the C326Y mutant FPN does not (absence of red dots) (Figure 6).

## Discussion

FPN is the only known iron exporter and thus is an important molecule in maintaining iron homeostasis [6]. Understanding the biology of FPN is essential in order to explain the molecular basis of disease caused by ferroportin mutations and to design and implement effective therapeutics targeting FPN. Although we have known about the importance of FPN for almost two decades, there are still some controversies surrounding the biology of the protein. One of these was the mechanism of degradation of FPN after hepcidin binds to it. Initial studies suggested that this process is mediated by the phosphorylation of FPN tyrosine residues 302 and 303 by the JAK-STAT signalling [42], but recently it was proven that instead, two lysine residues are the targets of ubiquitination and are required for effective internalization and degradation of FPN [1].

The membrane organization and topology of FPN has also been at the centre of controversy where different studies have shown varying results, showing localization differences between the N- and C-termini of the protein, the number of transmembrane domains, and whether FPN can form multimers [26,27,32–35]. Recently a structure for FPN was proposed based on a bacterial homologue where it was suggested that FPN undergoes conformational changes when exporting iron, and it is only when the protein is in an extracellular open configuration that hepcidin can bind to it [29]. Based on comparisons between macrophages from FD patients and healthy donors, it was suggested that FPN does not need to form dimers in macrophages and is iron-export competent (although iron export in macrophages from FD patients was reduced as compared with controls) [38]. The authors also suggested that in excess iron conditions, mutant FPN fails to reach the surface hence leading to iron overload, suggesting that it was the trafficking defect in excess iron conditions that was the pathological cause of the disease [38]. These models are able to explain how the protein functions as a monomer, but they do not explain whether it was the mutant or the WT protein that reached the surface. Research from our group and others have shown that although a mutant FPN (p.A77D and p.V162del mutations) is expressed on the cell surface, it is iron export incompetent [25,30].

In the present study, we examined the surface and intracellular expression of a number of WT and mutant FPN constructs using intracellular and surface immunofluorescence and a novel FPN antibody. We were able to show that both the N- and C-termini of the FPN protein have an intracellular localization. Interestingly, our antibody can detect only surface FPN expression and we suspect that this is due to the epitope that the antibody binds, which may be blocked while the protein is being trafficked. As suggested in the bacterial FPN model, the conformation of the protein changes, when it is exporting iron on the surface of the cell and this may result in exposure of epitopes that are hidden during trafficking of the protein. Using the relatively new approach of proximity ligation assays, we were able to show for the first time that FPN forms dimers and they can be localized in the cells. This is important in understanding the molecular basis of heterozygous mutations which cause trafficking defects in FPN. Mutant FPN protein molecules defective in trafficking bind to the WT protein and affect their proper localization and hence iron transport. We are also able to detect intracellular FPN and hepcidin interactions in cells transfected with both FPN and hepcidin constructs. This is the first time that these interactions have been observed *in situ* and the detection of these interactions suggests that when both hepcidin and FPN are expressed in the same cell, they can interact. These results have implications on our understanding of how FPN mediates iron export and the effect of mutations on the function of FPN.

A limitation of the present study is the use of exogenous overexpression models that can result in experimental artifacts, but most of our knowledge about the functioning of various iron homeostasis proteins comes from examining cellular functions using overexpression models. The primary reason for this is the absence of cell lines that express sufficient levels of detectable FPN protein, which emphasizes the need to use exogenous proteins. However, using PLA in the present study we were able to examine protein interactions in their native form in cells, unlike co-immunoprecipitation where lysing the cells can bring together proteins that would normally never come in contact and lead to false negatives and positives. The results from the present study will add important knowledge to the physiology of iron homeostasis and FPN biology in general, which will be helpful in designing therapeutics aimed at modulating FPN expression.

## Data Availability

Data sharing is not applicable to this article as no datasets were generated or analysed during the current study. All data generated or analysed during this study are included in this published article.

## Abbreviations

FAC: ferric ammonium citrate
FCS: fetal calf serum
FD: ferroportin disease
FPN: ferroportin
HH: hereditary hemochromatosis
TBST: Tween 20 in Tris-buffered saline
